# Breaking Procrastination Down: Differential Effects of Subtasking and Reinforcement on Engagement and Persistence

**DOI:** 10.3390/bs16101832

**Published:** 2026-10-07

**Authors:** Anusha Garg, Jonathan W. Schooler

**Affiliations:** Department of Psychological and Brain Sciences, University of California Santa Barbara, Santa Barbara, CA 93106, USA

**Keywords:** procrastination, temporal decision model, intervention, learned industriousness, reinforcement

## Abstract

Procrastination often reflects in-the-moment decisions about whether to start or continue working on a task, yet how brief interventions shift these decisions remains poorly understood. The present study examined whether task structuring and immediate reinforcement influence state procrastination through different mechanisms. Participants (*N* = 238) identified a real task they had been delaying and completed a control, subtask-only, or subtask-plus-reward condition. During a 15 min work period, they reported their engagement and the desire to continue, followed by measures of task appraisal and effort valuation. ANOVA models showed that breaking a task into subtasks increased immediate engagement relative to control, but adding reinforcement did not increase engagement further. Instead, reinforcement increased willingness to persist and the extent to which effort itself felt rewarding, and in a mediation model, this increase in effort valuation statistically accounted for the association between reinforcement and willingness to persist. These effects occurred without changes in task aversiveness or mood, and trait conscientiousness predicted persistence through the same effort–valuation pathway. Initiation and persistence thus appear to reflect distinct motivational processes: structuring supports getting started, whereas reinforcement sustains effort by making effort feel more rewarding.

## 1. Introduction

Putting off sending a dreaded email, writing a report, or studying for an exam until the last possible moment is a familiar experience for many. Procrastination is defined as the voluntary delay of an intended action despite expecting negative consequences (Steel, 2007). It has been associated with academic, occupational, financial, mental health, and physical health difficulties (Beutel et al., 2016; Fernie et al., 2017; Kroese et al., 2016; Rozental et al., 2022; F. M. Sirois, 2007, 2014, 2015). However, much of this research concerns chronic, trait-like patterns of procrastination. Less is known about state procrastination: the situational processes that produce delay at a particular moment.

State procrastination varies considerably within individuals across times, tasks, and situations (Bäulke et al., 2021; Wieland et al., 2018). It is shaped by social and environmental context (Koppenborg & Klingsieck, 2022) and reflects both trait- and state-level influences (Beck & Jackson, 2022; Koppenborg et al., 2024). Identifying the processes operating at moments of delay is therefore important both for understanding how procrastination unfolds and for designing brief interventions that can interrupt it as it occurs (Garg et al., 2025).

These moment-to-moment processes have an important but underexamined implication for intervention. Although a procrastination episode is typically framed as a failure to begin, initiating an avoided task and sustaining effort on it are distinct challenges. Even after starting, motivation may lapse, attention may drift, and delay may resume. Thus, an intervention that increases task engagement may not be the same as one that supports persistence, yet brief procrastination interventions have rarely been designed or evaluated with this distinction in mind. The present study addresses this gap by testing whether two intervention strategies differentially affect task engagement and persistence. Additionally, we examined state procrastination as a domain-general phenomenon to assess the impact of the intervention in broad contexts. In doing so, the study advances a more temporally differentiated account of a procrastination intervention—one that asks not only whether a strategy reduces delay, but also which stage of task engagement it supports.

### 1.1. Theoretical Framework

Three theoretical frameworks inform the present account of state procrastination: the Temporal Decision Model (TDM; Zhang & Feng, 2020), Temporal Motivation Theory (TMT; Steel & König, 2006), and the Theory of Learned Industriousness (Eisenberger, 1992). TDM and TMT are expectancy-value accounts. They model the in-the-moment decision of whether to begin acting now or to delay, holding that this choice depends on the relative costs and benefits of acting, with delayed outcomes weighing less than immediate ones. Learned Industriousness offers a complementary, reinforcement-based account. When effort has repeatedly been followed by reinforcement, effort itself may acquire secondary reward value. Together, these frameworks point to two potentially separable routes through which a brief intervention might reduce procrastination. Structuring a task into a small, actionable step may alter the appraisals that determine whether engagement begins. Reinforcing effort may instead increase the likelihood that engagement continues once underway.

This distinction is consistent with broader models of goal pursuit. The Rubicon Model of Action Phases separates the deliberative processes of choosing a goal from the volitional processes of initiating and carrying it out (Achtziger & Gollwitzer, 2018). Procrastination is characteristically a volitional failure: the intention to act is already formed, yet action has not begun. Even within this volitional phase, beginning an action and sustaining it are related but distinguishable challenges. The TRIALS framework makes a similar point. It holds that goal pursuit involves initiating, maintaining, and completing a course of action, and that each stage can fail in a different way and call for a different remedy (Sheeran & Webb, 2016). Procrastination likewise has been characterized as a self-regulatory failure involving difficulty initiating aversive goals (Van Eerde, 2000). Together, these frameworks suggest that an intervention that helps a person begin an avoided task may not necessarily help them persist with or complete it. Although this stage distinction is well-established in the goal-pursuit literature, it has rarely been examined within procrastination itself, where interventions have typically been directed at and evaluated against the initial decision to act.

These stage-specific processes unfold in the moment and are sensitive to fluctuating emotions and task appraisals. People may delay action to obtain short-term relief from negative affect (F. Sirois & Pychyl, 2013). Delay may also depend on the perceived value and temporal distance of task outcomes (Steel & König, 2006), and on the balance between a task’s immediate aversiveness and its anticipated utility (Zhang & Feng, 2020). Because these factors vary across situations, a growing literature has begun to examine state-level interventions delivered at or near the moment of delay (Garg et al., 2025; Koppenborg et al., 2024). Relevant approaches include implementation intentions (Gollwitzer, 1999), real-time self-regulation prompts (Svartdal et al., 2020), and brief affect-focused techniques (F. Sirois & Pychyl, 2013). Many, however, have been evaluated using trait measures of procrastination or without asking participants to act on a specific, currently avoided task. Little is therefore known about how brief interventions affect the immediate appraisals and behaviors underlying state procrastination.

Garg et al. (2025) addressed this question using a brief intervention grounded in the TDM (Zhang & Feng, 2020). In a large online experiment (*N* = 1035), participants identified a real task they were currently procrastinating on. They then completed a short sequence involving affect labeling (Torre & Lieberman, 2018), subtask generation, and the selection of an anticipated reward. Relative to two control conditions, the intervention increased perceived outcome utility and improved mood. It also widened the gap between outcome utility and task aversion, primarily through greater perceived utility, and raised participants’ reported likelihood of acting on the task. Changes in the utility–aversion balance, and to a lesser extent mood, partially accounted for the increase in behavioral intentions. These findings indicate that a brief intervention can shift appraisals associated with momentary task avoidance.

Two questions nevertheless remained unresolved. First, the intervention bundled affect labeling, subtask generation, and reward, so its effects cannot be attributed to any single component. Affect labeling did not reduce task aversion or stress, which leaves subtask generation and reward as the more plausible contributors. Isolating them is necessary both to refine the intervention and to determine whether they operate through different mechanisms. Second, the study measured participants’ reported likelihood of acting but gave them no opportunity to engage with the task itself. Intentions predict behavior, yet account for only a limited share of actual follow-through (Sheeran, 2002). The intervention may therefore have changed how motivated participants felt without changing whether they actually began or sustained their tasks.

One possibility is that subtask generation alone produced the observed improvements. Breaking a larger task into a small, concrete action can provide a clearer starting point, reduce perceived complexity, and make progress feel more attainable. Proximal subgoals can increase self-efficacy and motivation by providing more immediate indicators of progress (Bandura & Schunk, 1981; Huang et al., 2017), while simplifying the next required action can reduce planning demands and cognitive load (Maisto et al., 2015). Within the TDM, generating a proximal subtask may also improve the balance between anticipated utility and task aversiveness by creating a near-term, achievable goal that feels easier to begin (Zhang & Feng, 2020). Subtask generation also shares features with implementation intentions insofar as both make the next intended action more concrete (Gollwitzer & Sheeran, 2006). It could therefore improve task initiation even without an added reward.

A second possibility is that reward contributes benefits beyond those of subtasking. Its contribution, though, depends on how the reward operates. In Garg et al. (2025), participants selected an anticipated reward. This manipulation plausibly works through expected utility. TMT and TDM hold that procrastination becomes more likely when the benefits of completion are delayed and therefore discounted. Attaching an anticipated reward to a subtask raises the short-term utility of working and partly offsets the pull of more immediately gratifying alternatives (Duckworth et al., 2016). On this account, reward makes initial engagement more attractive by changing the immediate incentive structure.

Contingent reinforcement may operate differently. Here, reward is delivered after effort rather than merely anticipated, and it may therefore influence whether effort continues once underway. Learned Industriousness proposes that repeatedly reinforcing effort can cause effort itself to acquire secondary reward value (Eisenberger, 1992). A single intervention is unlikely to establish such an association from scratch. However, participants may enter the intervention with prior learning histories in which effort and progress have been followed by accomplishment, feedback, or reward. Previously acquired reward associations can shape later behavior when similar actions again predict rewarding outcomes (Wassum & Izquierdo, 2015; Williams, 1994). Extending this logic to the present context, delivering reinforcement contingent on subtask completion may reactivate existing effort–reward associations and increase the motivational value of continued effort. Reinforcement could therefore support persistence through a mechanism distinguishable from the anticipated utility emphasized by temporal decision models. Accordingly, the present study treats participants’ momentary perceptions of productivity as rewarding as an indicator of the reward value associated with effort, rather than as evidence that a new effort–reward association was acquired during a single session.

The present study addresses both the component and intention-only limitations of this earlier work. It experimentally separates subtask generation from the contingent reinforcement of subtask completion, allowing the effects of subtasking alone to be distinguished from the additional effects of reward. It also gives participants an opportunity to work on a real task they have been avoiding and assesses their reported engagement and follow-through across successive stages of task pursuit rather than assessing intentions alone. This design enables us to test whether subtask structuring supports initiation and contingent reinforcement supports persistence, and whether they do so through the distinct mechanisms outlined above: appraisal change for the former, the reward value of effort for the latter. The specific design and predictions are described below.

### 1.2. The Present Study

The present study examined whether a subtask-based intervention and contingent reinforcement differentially influence engagement and persistence during state procrastination. Participants identified a real task they had been procrastinating on and completed one of three conditions: Control, Subtask Only, or Subtask + Reward. Participants in both subtask conditions first completed the same brief recall exercise intended to evoke prior experiences of effort, progress, and reward, and then divided their task into smaller, actionable steps. Those in the Subtask + Reward condition additionally received brief positive feedback contingent on completing each subtask. Because the two active conditions shared the recall and subtasking procedures, their comparison isolated the additional effect of contingent reinforcement.

Participants were given an opportunity to work on their task and subsequently reported their engagement and willingness to persist. Midway through the task period, participants reported how actively they had been working and their desire to continue. Participants who did not complete the task during the 15 min period were instructed to message the experimenter when they subsequently completed it. The study also assessed participants’ enjoyment of effort and perceived reward from being productive, allowing us to examine whether changes in how effort was experienced could account for the effects of reinforcement.

First, we hypothesized that the subtask-based interventions would increase immediate task engagement relative to the control condition by providing a clear, proximal starting point, consistent with the expectancy-value accounts in which a proximal, achievable step improves the appraised balance of utility and aversiveness. Second, we expected contingent reinforcement to provide an additional benefit beyond subtask generation alone. Specifically, participants in the Subtask + Reward condition were expected to report greater enjoyment of effort, greater perceived reward from being productive, and a greater desire to continue working than participants in both the Subtask Only and Control conditions. They were also expected to be more likely to continue working when asked whether they would prefer to stop and to report completing their task within 24 h. This would be consistent with learned industriousness theory, in which reinforced effort acquires reward value.

Third, we predicted that perceived reward from effort would mediate the effect of condition on persistence and subsequent task completion. Such a pattern would be consistent with the proposition that reinforcement supports continued engagement by making effort itself feel more rewarding, rather than solely by increasing the anticipated utility of task completion.

Finally, because chronic procrastination has been consistently linked to lower trait conscientiousness (Steel, 2007), we also measured conscientiousness and included it as a covariate in our analyses. This allowed us to evaluate whether condition effects on engagement and persistence held above and beyond this well-established individual-difference correlate of procrastination. See Figure 1 for the conceptual model of the hypotheses.

## 2. Materials and Method

### 2.1. Participants

The study used a three-condition, between-subjects design: Control, Subtask Only, or Subtask + Reward. Each condition was administered online through separate Qualtrics surveys, linked to concurrently fielded Prolific studies. The aim was to recruit 100 participants per condition. Eligible participants resided in the United States, reported English as their first language, and were recruited through Prolific’s standard sampling procedures; no additional prescreen criteria were used. Additionally, filters were applied such that participants who completed one of the conditions were not eligible to participate in either of the other two conditions. The study descriptions on Prolific for all three conditions were the same. Participants thus selected among the concurrently available three conditions. The final number of entries Prolific noted was 379, given that it reopens recruitment when participants returned or did not complete a study, only closing recruitment when the 100 participant criterion per condition was met.

Participants were excluded in a stepwise manner. First, participants who did not provide informed consent were excluded (*n* = 10). Second, participants who did not complete 100% of the study were removed (*n* = 60). Third, participants were excluded if they reported a time spent on their selected task of less than 10 min or more than 20 min, reflecting a ±5 min window around the intended 15 min task duration (*n* = 67). This window was intended to exclude participants with poor task selection or inattentive responding. To evaluate whether this exclusion criterion influenced the results, we reran the primary condition analyses with these 67 participants included; the pattern of results was unchanged (see Supplementary Materials Section S1).

Fourth, all participants completed a seriousness check, which asked them to indicate whether they had responded attentively or had simply clicked through the survey. The prompt reassured participants that they would be compensated regardless of their response to encourage honest reporting. No participants indicated that they did not complete the study seriously; thus, no exclusions were made based on the seriousness check. Finally, participants whose written task responses consisted of gibberish or were otherwise unintelligible were deemed ineligible (*n* = 4).

After applying all exclusion criteria, the final analytic sample consisted of 238 participants: 79 in the Control condition, 82 in the Subtask Only condition, and 77 in the Subtask + Reward condition. The sample size was determined by budget constraints, with each participant receiving monetary compensation for completing the study. A post hoc sensitivity power analysis indicated that with the achieved group sizes, a one-way ANOVA with three groups had 80% power to detect effects of Cohen’s *f* ≥ 0.20 (equivalent to partial *n* ≥ 0.04) at *α* = 0.05.

The mean age of the final sample was 40.62 years (*SD* = 12.02). Of the participants, 55.7% identified as female, 43.9% as male, and 0.4% preferred not to report their gender. The racial composition of the sample was 70.7% White, 11.8% Black, 7.3% Mixed race, 6.5% Asian, and 3.3% Other. All participants resided in the United States. Additionally, 6.5% of the sample identified as students.

This study was approved by the University of California, Santa Barbara Human Subjects Committee (IRB approval number: 243-25-0527). The study was not preregistered.

### 2.2. Measures

**International Positive and Negative Affect Schedule-Short Form (I-PANAS-SF).** Participants completed the I-PANAS-SF (Thompson, 2007), a 10-item measure of mood and feelings and includes two subscales: the positive and negative attribute subscales. Each subscale contains five positive and negative markers of emotion, measured on a five-point Likert scale (*1 = very slightly/not at all–5 = extremely*), in accordance with items that represent affect like “Inspired”, and “Upset”. The scale was modified from the original version to assess mood in the moment as opposed to as a general tendency. Internal consistency in the present sample was *α* = 0.89 for the positive affect subscale and *α* = 0.87 for the negative affect subscale pre-task administration. Post-task was *α* = 0.88 and 0.90 respectively.

**Learned industriousness.** In classic learned industriousness research, Eisenberger and colleagues manipulated the effortfulness and reinforcement of specific laboratory or classroom tasks (e.g., anagram solving, math work, copying tasks) and indexed learned industriousness behaviorally via persistence, time on task, and performance on subsequent transfer tasks, rather than through self-report scales. In the present study, the construct was instead operationalized via self-report in the context of a task each participant had been avoiding (rather than a single generic laboratory task). Participants completed three items designed to assess the extent to which effort itself was experienced as positively valued during that personally relevant task (e.g., “I enjoyed putting effort into this task,” “I put a lot of effort into this task,” “Being productive during this task felt rewarding”), using a 7-point Likert-type scale ranging from *1 (strongly disagree) to 7 (strongly agree)*. These items were constructed to capture the core theoretical claim that, when effort has acquired secondary reward value, individuals experience working hard on a task as intrinsically rewarding, even when the task is one they would typically avoid. These three items demonstrated good internal consistency (*α* = 0.79).

Additionally, as a sensitivity check, we formed a reduced two-item composite restricted to the items that most directly index the reward value of effort (“I enjoyed putting effort into this task”; “Being productive during this task felt rewarding”), setting aside “I put a lot of effort into this task,” which reflects effort expenditure rather than its perceived reward value. Because alpha is inappropriate for two items, reliability was assessed with the Spearman–Brown coefficient = 0.84 (*r* = 0.72).

**Dormant learned industriousness.**

***Script.*** Participants in the Subtask Only and Subtask + Reward conditions were prompted to read this script: “Take a moment to think about a time when you had a list of tasks to complete, maybe for school, work, or even chores. Picture yourself crossing off each item after you complete it, one by one. Recall the burst of satisfaction and happiness with each task crossed off the list, and the feeling of progress. Notice how that sense of accomplishment builds, making it easier to keep going. Keeping that feeling of motivation when you’re in the flow in mind, please respond to the following questions.” This script was administered to both subtask conditions to hold constant activation of prior effort–reward associations across these groups.

***Manipulation check.*** 3 self-report questions were asked of the participants in the Subtask Only and Subtask + Reward conditions. This was meant to assess whether the recall prompt activated prior experiences of effort being followed by progress or reward. The items included “How rewarding did it feel to complete each subtask during the 15-min period?”, “How much did you experience a growing sense of motivation or progress as you completed each step of your task?”, and “As you worked through your subtasks, how strongly did you feel the satisfaction of making progress?”. They were all rated on a Likert scale of *1–7*, with anchors of *‘not at all’ to ‘extremely’*.

**NEO-Conscientiousness Scale.** The 60-item NEO-FFI (McCrae & Costa, 2004) assesses individual differences in 5 personality factors: Neuroticism, Extraversion, Openness to Experience, Conscientiousness, and Agreeableness, evaluated with 12 items each. For the purposes of the current study, we only evaluated the conscientiousness scale. All items were measured on a five-point Likert scale ranging from *(1) strongly disagree to (5) strongly agree*. Example items from the conscientiousness subscale include “I keep my belongings neat and clean”, and “I’m pretty good about pacing myself so as to get things done on time”. Internal consistency in the present sample was *α* = 0.91.

### 2.3. Procedure

After providing informed consent, participants were asked to identify a task they were currently procrastinating on or anticipated procrastinating on that could realistically be completed within approximately 15 min (see Figure 2 for a schematic overview of the full procedure). Participants were instructed to select a task that they could, in principle, begin working on immediately while completing the study. They then reported an estimated time required to complete the task.

As described above, participants completed one of three conditions—Control, Subtask Only, or Subtask + Reward—depending on which of the concurrently available studies they accessed. Participants in the Subtask Only and Subtask + Reward conditions were prompted to first read the dormant learned industriousness script (see the Section 2.2). Participants in the Control condition did not receive this prompt.

Next, participants in the Subtask Only and Subtask + Reward conditions were instructed to break their selected task into three to five subtasks, each expected to take approximately three to five minutes to complete, and to enter these subtasks into the survey interface. Participants in the Control condition did not engage in subtask planning and proceeded directly to the baseline measures.

All participants then completed baseline assessments of task aversiveness (“how unpleasant does doing the task feel to you?”) and mood through the PANAS. Following these measures, participants were instructed to work on their identified task for a total of 15 min. Before beginning, participants were prompted to gather any necessary materials and to ensure that the survey screen remained accessible throughout the task period.

During the 15 min work period, participants in the Subtask Only and Subtask + Reward conditions were guided through their task one subtask at a time. Participants advanced to the next subtask only after indicating that they had completed the current one. In the Subtask + Reward condition, participants received contingent positive reinforcement: brief performance-contingent congratulatory feedback presented on the screen immediately after each completed subtask (e.g., “You did it! Nice going!”). We use contingent positive reinforcement to denote this manipulation throughout, reserving congratulatory feedback for the specific on-screen stimulus. Participants in the Subtask Only condition advanced through their subtasks without receiving any reinforcing feedback. Participants in the Control condition were instructed simply to work on their task for the full duration, without subtask guidance or feedback.

At the midpoint of the work period (approximately 8 min), all participants were prompted to pause their work and complete a brief follow-up questionnaire assessing the extent to which they had been actively working on the task, and their desire to continue working on the task. Participants then resumed the task until the 15 min period concluded.

After completing the work period, participants completed additional self-report measures. Those in the Subtask Only and Subtask + Reward conditions completed the dormant learned industriousness manipulation check, then all conditions responded to the learned industriousness questionnaire, post-measure of task aversiveness and mood, and finally trait conscientiousness. Participants were also asked whether they had completed their task and to report the percentage of the task they had completed.

To assess behavioral follow-through beyond the experimental session, participants were instructed to contact the experimenter via Prolific messaging once they had fully completed their procrastinated task, whenever that occurred. Participants were informed that this message would serve as an important data point for the study but that compensation was not contingent on task completion.

Finally, all participants completed a seriousness check, were debriefed regarding the purpose of the study, and received $5 compensation for their participation.

## 3. Results

### 3.1. Analysis Strategy

All analyses were conducted in R, with *α* = 0.05 and two-tailed tests throughout. Missing data were handled using listwise deletion. Conscientiousness was included as a covariate in all relevant analyses to account for individual differences.

We first examined whether the active conditions—which combined a recall prompt, subtasking, and guided progression—increased engagement and persistence relative to control, and whether contingent reinforcement provided an incremental benefit beyond this structuring bundle. Condition differences on mid-task and post-task self-report outcomes were tested using one-way ANOVAs; significant omnibus effects were followed by Tukey-adjusted pairwise comparisons for the three-condition contrasts of interest (Subtask Only vs. Control, Subtask + Reward vs. Control, Subtask + Reward vs. Subtask Only).

A separate question concerned whether the experimental manipulation activated what it was designed to activate: participants’ prior associations between effort and reward. We assessed this via a three-item manipulation check, comparing scores using a one-way ANOVA between the Subtask + Reward and Subtask Only conditions. We also examined the association between this manipulation check and the post-task learned industriousness evaluation composite (effort enjoyment, perceived effort, and reward value of productivity) using a Pearson correlation, to assess the degree of convergence between activation of prior effort–reward associations and participants’ subsequent evaluations of their own effort.

As a secondary behavioral outcome, task completion (completed vs. not completed within the 15 min session) was compared across conditions using a chi-square test of independence, and the relation between mid-task engagement/willingness and completion was examined using Welch two-sample *t*-tests comparing completers and noncompleters; given the small number of noncompleters (*n* = 17), these comparisons have limited power. Completion rates were near ceiling across conditions, leaving too little outcome variance to estimate a mediation model of condition effects on completion via learned industriousness evaluation. We therefore do not report a formal mediation test of this pathway. Fourteen participants (5.9%) voluntarily messaged the experimenter after the session to report completing their task (6 Subtask + Reward, 4 Subtask Only, 4 Control), most within a few hours though two replied several days later. Because messaging was optional and unincentivized, these reports index the reporting of completion rather than completion itself: completion status is unknown for the participants who did not message, so the counts cannot be interpreted as condition differences in actual follow-through. We therefore report them descriptively and do not subject them to significance testing.

To characterize relations among the continuous study variables, we computed zero-order Pearson correlations.

Finally, to test whether learned industriousness evaluation accounted for condition effects on willingness to continue working, we estimated a multicategorical mediation model in lavaan, with condition dummy-coded relative to Control (Subtask Only and Subtask + Reward) predicting both the mediator and the outcome. All three pairwise comparisons, including Subtask + Reward vs. Subtask Only, were estimated as defined parameters within the same multicategorical model, and conscientiousness was included as a covariate consistent with the primary analyses. Indirect effects were estimated using nonparametric bootstrap standard errors (5000 resamples, with a fixed random seed for reproducibility as 20260815). Because the learned industriousness evaluation composite was measured after the 15 min work period ended, while willingness to continue was measured at the 8 min mid-task checkpoint, we note this measurement-timing constraint on how the indirect effects should be interpreted, and return to it in the Section 4.

Because one item in the learned industriousness composite indexed effort expenditure rather than effort valuation, we additionally reran the mediation using a composite restricted to the two valuation items, as a sensitivity analysis; this is reported alongside the primary model.

### 3.2. Task Descriptives

Participants named a task they were currently procrastinating on and could reasonably work on for approximately 15 min. The mean estimated task duration was 14.08 min (*SD* = 3.07). Tasks spanned a range of domains, including academic work, professional tasks, household chores, and personal or administrative activities, supporting the ecological validity of the task selection (Figure 3).

### 3.3. Manipulation and Baseline Checks

Baseline equivalence across conditions was first examined. Pre-task aversiveness and baseline positive and negative affect did not differ by condition, all *ps* > 0.10.

A manipulation check assessed whether the dormant learned industriousness script activated memories of past rewarding effort. This check was administered only to participants in the Subtask Only and Subtask + Reward conditions. Participants in the Subtask + Reward condition reported stronger perceptions of effort–reward activation (*M* = 5.60, *SD* = 1.32) than those in the Subtask Only condition (*M* = 4.99, *SD* = 1.77), *F*(1, 157) = 4.80, *p* = 0.030, *η*^2^ = 0.03. This suggests that while the recall prompt activated prior effort–reward memories in both subtask conditions, contingent reinforcement during task engagement further strengthened these associations.

### 3.4. Condition Differences for Mid-Task Evaluations and Task Completion Rates

To assess whether the experimental conditions differed significantly from the control condition, a series of one-way analyses of variance (ANOVAs) were conducted on self-reported measures related to participants’ procrastinated tasks. Condition differences were tested using one-way ANOVAs. Significant omnibus effects were followed by Tukey-adjusted pairwise comparisons for the three-condition contrasts of interest: Subtask Only vs. Control, Subtask + Reward vs. Control, and Subtask + Reward vs. Subtask Only. Including conscientiousness as a covariate did not alter the pattern of results for either of the mid-task questions. Although conscientiousness was a significant positive predictor of both mid-task evaluation outcomes (active task engagement and willingness to continue) (all *ps* ≤ 0.016), the main effects of Condition remained significant after controlling for conscientiousness.

**Task engagement.** During the 15 min work period, participants were interrupted at the 8 min mark and asked to report (a) how actively they had been working on the task and (b) how much they wanted to continue working.

***Active task engagement.*** Mean task activity was highest in the Subtask + Reward condition (*M* = 8.38, *SD* = 1.17), followed by the Subtask Only condition (*M* = 8.01, *SD* = 1.42), and lowest in the Control condition (*M* = 6.43, *SD* = 1.16) (Figure 4).

A one-way ANOVA revealed a significant main effect of condition on task activity, *F*(2, 235) = 53.14, *p* < 0.001, *η*^2^ = 0.31. Tukey’s HSD post hoc tests indicated that both experimental conditions produced significantly greater task activity than the Control condition. Participants in the Subtask + Reward condition reported higher activity than those in the Control condition (*mean difference* = 1.95, *p* < 0.001, *Cohen’s d* = 1.67, 95% *CI* [1.47, 2.42]), representing a large effect. Similarly, the Subtask Only condition exceeded the Control condition (*mean difference* = 1.58, *p* < 0.001, *d* = 1.22, 95% *CI* [1.11, 2.05]), also a large effect.

The difference between the two subtask conditions was not statistically significant (*mean difference* = −0.36, *p* = 0.16, *d* = 0.28). Taken together, these results indicate that the structured conditions substantially increased task activity relative to control, whereas the addition of contingent reinforcement did not significantly enhance activity beyond this benefit.

***Desire to continue working.*** Mean continuation scores were highest in the Subtask + Reward condition (*M* = 7.43, *SD* = 1.88), followed by the Subtask Only condition (*M* = 6.49, *SD* = 2.51) and the Control condition (*M* = 4.27, *SD* = 2.22) (Figure 5).

A one-way ANOVA revealed a significant effect of condition on willingness to continue working, *F*(2, 235) = 41.72, *p* < 0.001, *η*^2^ = 0.26. Tukey post hoc tests showed that both intervention conditions differed significantly from the Control condition. Participants in the Subtask + Reward condition reported higher continuation scores than those in the Control condition (*mean difference* = 3.16, *p* < 0.001, *d* = 1.54, 95% *CI* [2.32, 4.00]), reflecting a large effect. Participants in the Subtask Only condition also reported greater willingness to continue than those in the Control condition (*mean difference* = 2.22, *p* < 0.001, *d* = 0.93, 95% *CI* [1.39, 3.05]).

Importantly, participants in the Subtask + Reward condition reported significantly greater willingness to continue than those in the Subtask Only condition (*mean difference* = 0.94, *p* = 0.022, *d* = 0.43), indicating an additional benefit of adding contingent reinforcement to the structured task. These findings suggest that while both interventions increased motivation to continue relative to control, reinforcement provided an incremental boost beyond subtasking alone.

**Task completion.** Task completion was coded as a binary outcome (*1 = completed, 0 = not completed*) based on whether participants reported completing the task within the 15 min period. Completion rates were high across conditions: 91.1% (*n* = 72/79) in the Control condition, 92.2% (*n* = 71/77) in the Subtask + Reward condition, and 95.1% (*n* = 78/82) in the Subtask Only condition. A chi-square test of independence indicated that completion rates did not differ significantly across conditions, *χ*^2^(2, *N* = 238) = 1.03, *p* = 0.60. Thus, participants in the Subtask Only and Subtask + Reward conditions were not more likely to complete their tasks within the allotted time than participants in the Control condition.

To further examine the relation between engagement and completion, Welch two-sample *t*-tests compared participants who completed the task to those who did not. Participants who completed the task (*n* = 221) reported significantly higher task activity at the 8 min mark (*M* = 7.71, *SD* = 1.32) than those who did not complete the task (*n* = 17; *M* = 6.24, *SD* = 2.72), *t*(16.59) = 2.21, *p* = 0.041, 95% *CI* [0.06, 2.89].

In contrast, participants who completed the task (*M* = 6.12, *SD* = 2.58) did not report significantly greater willingness to continue than those who did not complete the task (*M* = 5.18, *SD* = 2.43), *t*(18.90) = 1.54, *p* = 0.14, 95% *CI* [−0.34, 2.23]. Although the direction of the effect was consistent with expectations, willingness to continue midway through the task did not reliably predict task completion.

### 3.5. Task Aversiveness and Affect

Participants completed pre- and post-task measures of task aversiveness and positive and negative affect. Change scores were computed by subtracting pre-task ratings from post-task ratings, such that negative values indicated reductions in aversiveness or negative affect and positive values indicated increases in positive affect.

**Task aversiveness.** Across all conditions, participants reported modest reductions in task aversiveness following task engagement (Control: *M* = −0.39, *SD* = 1.04; Subtask Only: *M* = −0.56, *SD* = 1.16; Subtask + Reward: *M* = −0.58, *SD* = 0.92). A one-way ANOVA revealed no significant differences in aversiveness change across conditions, *F*(2, 235) = 0.79, *p* = 0.46, *η*^2^ = 0.007, indicating that reductions in aversiveness did not differ as a function of subtask structuring or reinforcement. Including conscientiousness as a covariate did not alter this pattern. Neither condition nor conscientiousness significantly predicted change in aversiveness, and the Condition × Conscientiousness interaction was not significant (all *ps* ≥ 0.46).

**Positive affect.** Participants reported small increases in positive affect from pre- to post-task across conditions (Control: *M* = 2.16, *SD* = 2.95; Subtask Only: *M* = 2.65, *SD* = 3.00; Subtask + Reward: *M* = 2.87, *SD* = 3.50). Change in positive affect did not differ significantly by condition, *F*(2, 235) = 1.02, *p* = 0.36, *η*^2^ = 0.009. Although the Subtask + Reward condition showed the largest mean increase, this difference was not statistically significant. Controlling for conscientiousness did not change the results. Neither condition nor conscientiousness significantly predicted positive affect change, and the interaction was not significant (all *ps* ≥ 0.36).

**Negative affect.** Negative affect decreased modestly following task engagement across all conditions (Control: *M* = −0.86, *SD* = 2.35; Subtask Only: *M* = −1.16, *SD* = 2.20; Subtask + Reward: *M* = −0.97, *SD* = 2.11). A one-way ANOVA revealed no significant differences in negative affect change by condition, *F*(2, 235) = 0.37, *p* = 0.69, *η*^2^ = 0.003. When conscientiousness was included as a covariate, conscientiousness emerged as a significant predictor of negative affect change, *F*(1, 234) = 13.97, *p* < 0.001, indicating that individuals higher in conscientiousness experienced larger reductions in negative affect. The main effect of condition and the Condition × Conscientiousness interaction remained nonsignificant (both *ps* ≥ 0.19).

### 3.6. Learned Industriousness

Participants rated three items assessing the extent to which exerting effort during the task was enjoyable, effortful, and rewarding. The items demonstrated good internal consistency (*α* = 0.79) and were examined both individually and as an averaged composite. Dormant learned industriousness scores measured after the recall prompt were strongly correlated with this composite, *r* = 0.78, *p* < 0.001, indicating that the manipulation check and the composite outcome capture related but distinguishable constructs. Including conscientiousness as a covariate did not alter the significance or direction of the condition effects reported below.

#### Item Level Analyses

***Enjoyment of effort.*** Enjoyment of putting effort into the task differed by condition, *F*(2, 235) = 3.99, *p* = 0.020, *η*^2^ = 0.03. Participants in the Subtask + Reward condition reported greater enjoyment (*M* = 5.39, *SD* = 1.30) than those in the Control condition (*M* = 4.67, *SD* = 1.80; *mean difference* = 0.72, *p* = 0.017). The Subtask Only condition (*M* = 4.90, *SD* = 1.71) did not differ significantly from either condition (both *ps* > 0.14).

***Perceived effort.*** Perceived effort did not differ by condition, *F*(2, 235) = 0.08, *p* = 0.93, *η*^2^ < 0.01. Participants across all conditions reported comparable levels of effort expenditure during the task.

***Reward value of productivity.*** Condition significantly predicted how rewarding participants found being productive, *F*(2, 235) = 3.52, *p* = 0.031, η^2^ = 0.03. Participants in the Subtask + Reward condition (*M* = 6.14, *SD* = 0.87) reported greater reward value than those in the Control condition (*M* = 5.63, *SD* = 1.49; *mean difference* = 0.51, *p* = 0.042). No other pairwise comparisons reached significance.

***Composite learned industriousness.*** The composite learned industriousness score showed a marginal effect of condition after controlling for conscientiousness, *F*(2, 234) = 2.62, *p* = 0.075, *η*^2^ = 0.02. Participants in the Subtask + Reward condition tended to report higher composite scores than those in the Control condition (*mean difference* = 0.39, *p* = 0.098), whereas the Subtask Only condition did not differ significantly from either condition (both *ps* > 0.22).

***Effort-valuation composite (sensitivity).*** One item (“I put a lot of effort into this task”) indexes effort expenditure rather than the reward value of effort. As perceived effort did not differ by condition (see above), we also examined a composite restricted to the two valuation items (“I enjoyed putting effort into this task”; “Being productive during this task felt rewarding”; *r* = 0.72). On this composite, condition had a significant effect, F(2, 235) = 4.36, *p* = 0.014, which held with conscientiousness included, F(2,234) = 4.66, *p* = 0.01. Critically, the Subtask + Reward condition exceeded the Subtask Only condition on effort valuation (mean difference = 0.47, *p* = 0.031; covariate-adjusted *p* = 0.021). This contrast isolates the incremental effect of contingent reinforcement, because the recall prompt, subtasking, and guidance were held constant across the two active conditions. The full parallel results are reported in Supplementary Materials Section S2.

***Correlations.*** Table 1 reports the full correlation matrix among all continuous state measures. Both pre-task and post-task aversiveness were negatively correlated with participants’ evaluation of the task as rewarding (LI evaluation; *r* = −0.18, *p* < 0.01; *r* = −0.42, *p* < 0.001, respectively). Similarly, reductions in aversiveness across the task (change in aversion) were associated with higher reward evaluation (*r* = −0.23, *p* < 0.001). Motivation to continue working was in turn strongly correlated with how rewarding participants later found the task (*r* = 0.49, *p* < 0.001). Conscientiousness was positively correlated with finding the task rewarding (*r* = 0.30, *p* < 0.001) and with improvements in positive affect (*r* = 0.24, *p* < 0.001), and pre-task aversiveness was positively related to pre-task negative affect (*r* = 0.27, *p* < 0.001), consistent with an emotion-regulation account of task avoidance.

### 3.7. Mediation

To test whether learned industriousness evaluation (the composite measure of effort enjoyment, perceived effort, and reward value of productivity) accounted for condition effects on willingness to continue working, we estimated a single multicategorical mediation model in *lavaan* (Figure 6). Condition was dummy-coded relative to Control (Subtask Only, Subtask + Reward) and entered as a predictor of both the mediator and the outcome within one model estimated on the full sample (*N* = 238); the Subtask + Reward vs. Subtask Only contrast was obtained as a defined parameter within this same model. Conscientiousness was included as a covariate on both the mediator and the outcome. The model was estimated using nonparametric bootstrap standard errors (5000 resamples, fixed seed = 20260815).

Subtask Only did not significantly predict learned industriousness evaluation relative to Control, *a*_1_ = 0.09, *SE* = 0.19, *p* = 0.640, 95% bootstrap CI [−0.28, 0.46]. Subtask + Reward did significantly predict learned industriousness evaluation relative to Control, *a*_2_ = 0.41, *SE* = 0.17, *p* = 0.016, 95% bootstrap CI [0.09, 0.77]. Conscientiousness was itself a significant positive predictor of learned industriousness evaluation, but not of willingness to continue once learned industriousness evaluation was in the model (*ps* reported below). Learned industriousness evaluation was a strong positive predictor of willingness to continue across the full sample, *b* = 0.96, *SE* = 0.11, *p* < 0.001.

The indirect effect of Subtask Only (vs. Control) on willingness to continue through learned industriousness evaluation was not significant, *ab* = 0.09, *SE* = 0.19, *p* = 0.645, 95% bootstrap CI [−0.26, 0.46]. The indirect effect of Subtask + Reward (vs. Control) was significant, *ab* = 0.40, *SE* = 0.18, *p* = 0.024, 95% bootstrap CI [0.08, 0.78]. The indirect effect of Subtask + Reward relative to Subtask Only was at the significant boundary, *ab* = 0.31, *SE* = 0.16, *p* = 0.053, 95% bootstrap CI [0.01, 0.65]. As reported in the sensitivity analysis below, this effect is clearly significant when the mediator is restricted to the two effort-valuation items.

Direct effects of condition on willingness to continue remained significant for all three contrasts: Subtask Only vs. Control, *c′* = 2.15, *SE* = 0.30, *p* < 0.001, 95% bootstrap CI [1.54, 2.73]; Subtask + Reward vs. Control, *c′* = 2.79, *SE* = 0.29, *p* < 0.001, 95% bootstrap CI [2.18, 3.36]; and Subtask + Reward vs. Subtask Only, *c′* = 0.65, *SE* = 0.32, *p* = 0.043, 95% bootstrap CI [0.02, 1.28]. Total effects were significant for all three contrasts (Subtask Only vs. Control, *c* = 2.23, *SE* = 0.37, *p* < 0.001; Subtask + Reward vs. Control, *c* = 3.19, *SE* = 0.33, *p* < 0.001; Subtask + Reward vs. Subtask Only, *c* = 0.96, *SE* = 0.35, *p* = 0.006). The direct effect of conscientiousness on willingness to continue, once learned industriousness evaluation was in the model, was not significant (*p* = 0.945), indicating that conscientiousness’s association with persistence operates largely through its association with learned industriousness evaluation rather than through a separate direct pathway.

Together, these results show an interesting pattern. Subtask structuring increased willingness to persist directly, without evidence that this operated through increased perceived rewardingness of effort. Reinforcement, by contrast, increased willingness to persist directly and indirectly through increased learned industriousness evaluation. This indirect pathway was evident clearly for the Subtask + Reward vs. Control contrast and at the boundary of significance for Subtask + Reward vs. Subtask Only, becoming clearly significant when the mediator is restricted to the two effort-valuation items. Because the learned industriousness evaluation composite was measured after the 15 min work period ended, while willingness to continue was measured at the 8 min mid-task checkpoint, these models describe statistical association among condition, effort valuation, and persistence rather than establishing temporally ordered mediation; we return to this measurement-timing constraint in the Section 4.

**Sensitivity analysis (effort-valuation items only).** For the reasons noted in Section 3.6, we re-estimated the same multicategorical model using the two-item effort-valuation composite in place of the three-item composite, holding model specification, covariate, seed, and bootstrap procedure constant. Here, the Subtask + Reward versus Subtask Only indirect effect was significant on the two-item composite (*ab* = 0.41, *SE* = 0.17, *p* = 0.015, 95% bootstrap CI [0.10, 0.77]). This change was carried by the a-path (condition to effort-valuation), which strengthened from 0.32 to 0.49; the b-path was comparable across the two composites (0.96 and 0.85). The Subtask + Reward versus Control indirect effect was likewise larger on the two-item composite, *ab* = 0.55, *p* = 0.002, 95% bootstrap CI [0.22, 0.92], and the Subtask Only versus Control indirect effect remained nonsignificant in both composites (three-item *p* = 0.65; two-item *p* = 0.51). Correspondingly, the Subtask + Reward versus Subtask Only direct effect declined from significant on the three-item composite (*c’* = 0.65, *p* = 0.043) to nonsignificant on the two-item composite (*c’* = 0.54, *p* = 0.086), while the total effect was unchanged by construction (*c* = 0.96, *p* = 0.006). Full results appear in Supplementary Materials Section S2.

## 4. Discussion

Starting an avoided task and staying with it are not the same problem, and the present findings suggest that they may respond to different intervention components. Procrastination is often conceptualized as an individual difference, yet many instances emerge from moment-to-moment decisions about whether to engage with and persist on aversive or effortful tasks. Building on prior work showing that brief, in-the-moment interventions can shift motivation to work on procrastinated tasks (Garg et al., 2025), the present study examined how such interventions operate. Garg et al.’s intervention increased intentions to work on procrastinated tasks but did not disentangle whether subgoal generation or reinforcement was primarily responsible for these effects. The present findings indicate that these components influence state procrastination through distinct but complementary motivational pathways. Relative to the Control condition, both the Subtask Only and Subtask + Reward conditions produced significantly greater mid-task activity, indicating that the structured intervention—which combined a recall prompt, subtasking, and guided progression—increased immediate engagement. In contrast, the direct comparison between the two active conditions showed that reinforcement increased willingness to continue working and showed a trend toward increasing the perceived rewardingness of effort. Together, these contrast-specific findings indicate that the structured interventions primarily facilitate initial engagement, whereas reinforcement contributes additional effects on persistence potentially by changing how effort is valued once work is underway. This pattern is consistent with a staged view of goal pursuit: the structuring package acts on engagement with an avoided task, while reinforcement acts on its continuation—separable challenges that procrastination research has increasingly treated as distinct (Svartdal et al., 2020).

Consistent with predictions of the Temporal Decision Model (Zhang & Feng, 2020), the structured interventions robustly increased immediate task engagement. Particularly, subtask creation is the component most directly linked to the TDM. Importantly, unlike Garg et al. (2025), which assessed intentions before task engagement, the present study captured participants’ engagement as they worked on a personally avoided task, showing that subtask structuring can influence involvement during task performance, rather than merely intentions to begin. Participants in both intervention conditions reported higher mid-task activity than participants in the Control condition, a difference that emerged at the point of doing the task, not in anticipation of it. Within the TDM framework, this effect may reflect a shift in engagement utility. Breaking a task into proximal, achievable steps reduces uncertainty about how to begin, lowers cognitive and planning demands, and provides immediate cues of progress. Together, these changes may make the immediate value of engaging outweigh the short-term appeal of continued delay. This, paired with the industriousness recall prompt, led to the two active intervention conditions displaying greater task engagement than the control condition.

A different pattern emerged for the desire to continue working. Participants who received reinforcement after completing subtasks reported greater desire to persist than those who generated subtasks alone, even though both conditions showed comparable levels of mid-task activity. Additionally, post-task evaluations showed that reward selectively increased enjoyment of exerting effort and the perceived reward value of being productive. In a multicategorical mediation model, increased effort valuation carried a significant indirect effect from reinforcement to willingness to continue relative to Control. The three-item composite yielded an indirect effect at the boundary of significance (*p* = 0.053). The two-item composite, which captures the reward value of effort more directly, yielded a clearly significant indirect effect for the Subtask + Reward versus Subtask Only contrast (*p* = 0.015). Additionally, on the construct-focused measure the residual direct path was no longer significant while the total effect was unchanged, consistent with effort valuation accounting for more of this association. This pattern suggests that reinforcement does not simply amplify task initiation and engagement. Instead, it may alter how effort itself is evaluated once engagement has begun. Effort valuation was measured after the work period rather than at the mid-task checkpoint, so these models establish that condition, effort valuation, and persistence were associated rather than establishing a strict temporal sequence. Nonetheless, the pattern across the manipulation, mediator, and outcome is consistent with the account that reward sustains engagement by making effort itself feel more rewarding. Critically, the two routes appear mechanistically distinct: the structured intervention raised engagement but did not act through effort valuation, whereas reward’s effect on persistence did. Expectancy-value accounts such as the Temporal Decision Model and Temporal Motivation Theory capture initiation well—restructuring a task changes the near-term costs and benefits of starting—but they do not, on their own, explain why persistence rose specifically as effort came to feel more rewarding. Accounting for that shift calls for a reinforcement-based mechanism operating alongside expectancy-value processes.

This interpretation is consistent with learned industriousness theory (Eisenberger, 1992), which proposes that effort can acquire secondary reward value when consistently paired with reinforcement. Whereas classic learned industriousness paradigms rely on repeated reinforcement across extended training to produce durable shifts in effort valuation, the present findings suggest a more immediate process. Rather than demonstrating new learning within a single session, the results are more consistent with the increased salience and re-engagement of previously established effort–reward associations. Most individuals have prior histories in which effortful engagement has been followed by progress feedback or symbolic reinforcement. By delivering contingent feedback during subtask completion, the present manipulation may have activated these preexisting associations, leading effort to be experienced as more rewarding in the moment and thereby supporting continued engagement. This shift was specific to how rewarding effort felt: the amount of effort participants reported expending did not differ across conditions. Reinforcement did not make participants work harder, but may have made the effort they exerted feel more worthwhile—a revaluation of effort consistent with learned industriousness theory, rather than a simple increase in exertion. Notably, although a brief recall prompt activated prior effort–reward memories in both subtask conditions, only contingent reinforcement during task engagement produced significant increases in the perceived rewardingness of effort relative to the Control condition. The manipulation check, administered after the work period, itself was sensitive to contingent reinforcement, such that participants in the Subtask + Reward condition reported stronger perceptions of effort–reward activation than those in the Subtask Only condition, showing that contingent reinforcement during task engagement further strengthened these associations. This pattern suggests that contingent reinforcement, rather than the recall prompt alone, was necessary to re-engage these associations strongly enough to influence the momentary valuation of effort.

The same account may also help explain individual differences. Trait conscientiousness predicted greater willingness to persist, but this relationship operated largely through effort valuation: once the perceived rewardingness of effort was accounted for, conscientiousness no longer independently predicted persistence, and more conscientious participants also found being productive more rewarding. This is consistent with the possibility that people who habitually follow through carry richer histories of effort being reinforced, and it suggests that the state engaged by our manipulation and the trait captured by conscientiousness may be linked through a common underlying process: the degree to which effort has acquired reward value. On this reading, the intervention briefly induces a state that conscientious individuals may occupy more readily. This individual difference pathway operated alongside, and separately from, condition: Conscientiousness predicted larger reductions in negative affect even though condition did not, indicating that the trait shaped the affective experience of the task through a route the brief manipulation did not engage. This convergence is compatible with personality research: industriousness and low procrastination are empirically established facets of the same trait, conscientiousness (MacCann et al., 2009), and the effortful, persistent side of that trait has developmental origins in self-regulation and academic motivation (Eisenberg et al., 2014). Learned industriousness supplies a reinforcement-based account of how effort comes to carry this value (Eisenberger, 1992). The current results extend that account to the state level, with in-the-moment reinforcement reproducing a pattern of effort–reward valuation that more conscientious individuals may be more likely to bring to the task.

The present findings also clarify the distinction between task aversiveness and the rewardingness of effort. Although these constructs were moderately correlated, they showed different sensitivity to the experimental manipulations. Neither subtask structuring nor reinforcement produced differential changes in pre–post-task aversiveness or mood relative to the Control condition. In contrast, reinforcement increased the extent to which exerting effort and being productive were experienced as rewarding in post-task evaluations. As a result, motivation and persistence increased despite stable levels of task aversiveness across conditions. This dissociation suggests that task aversiveness and effort rewardingness reflect related but distinct components of task appraisal. This pattern mirrors prior work showing that state-level interventions can increase outcome utility without reducing task aversion (Garg et al., 2025) and aligns with findings that increases in task–outcome value, but not decreases in aversiveness, predict reductions in procrastination behavior (Chen et al., 2025). Extending this work, the present results indicate that value-based routes to reduced procrastination can operate not only through enhanced outcome value but also through increased rewardingness of effort, even when task aversiveness remains unchanged. Thus, sustaining engagement may depend less on making an unpleasant task feel less aversive than on making continued effort feel more worthwhile.

Taken together, these findings provide a more precise account of how brief, state-level interventions influence procrastination. Task structuring appears sufficient to facilitate early engagement by lowering barriers to action. Reinforcement, in contrast, selectively enhances persistence by increasing the perceived value of effort. This extends and refines Garg et al. (2025): whereas their intervention paired subtasking with an anticipated future reward, the present results suggest that delivering reward contingently as effort unfolds can help sustain engagement above and beyond the structured interventions alone. Theoretically, the current study advances models of state procrastination by separating engagement from persistence. Procrastination accounts have largely treated momentary delay as a failure to begin. The present dissociation places task structuring and contingent reinforcement at different stages of task pursuit, and identifies the reward value of effort as a mechanism specific to persistence. This work informs the design of scalable interventions that target the specific motivational processes underlying momentary delay.

Practically, initiation and persistence call for different supports: a tool that only helps users break a task down should ease initial engagement but may be insufficient to keep them going, whereas sustaining engagement may require delivering reward or feedback as effort unfolds. The right support may also depend on the kind of task. Participants here worked on self-selected tasks from academic, occupational, household, and personal domains (Figure 3). While matching support to task type is outside of the scope of the current manuscript, future work that manipulates task type directly could determine which supports suit which tasks. Because these effects emerged in a nationally distributed adult sample rather than an undergraduate sample, which remains common in procrastination research, they offer insight into how such supports might function in the everyday, non-academic settings.

### Limitations and Future Directions

Although the present study clarifies how task structuring and reinforcement influence state procrastination, several limitations should be noted. First, although task completion within the 15 min window did not differ across conditions, robust differences emerged at the midpoint in both reported activity and willingness to continue working. The high overall completion rate (91–95%) likely restricted variability and reduced sensitivity for detecting between-condition differences in final completion. Participants were instructed to engage with their procrastinated task during the 15 min timer, and most complied with this instruction. As a result, the structured work period may have constrained opportunities for differential initiation or disengagement across conditions. Of the participants who did not finish within the timer, 14 later voluntarily messaged the experimenter to report completing their task (6 in Subtask + Reward, 4 in Subtask Only, and 4 in Control). The present evidence therefore speaks to motivation during the task rather than to eventual completion. Future studies could use more flexible or open-ended work periods and scheduled follow-up assessments to permit a more sensitive test of how subtask structuring and reinforcement influence spontaneous initiation and sustained persistence.

Second, the primary outcomes were self-reported by unsupervised online participants, and compliance with the reflection prompt and the full work period could not be directly verified. There is little reason to attribute the results to demand characteristics, however: reinforcement moved specific outcomes—enjoyment of effort, the reward value of productivity, and willingness to persist—while leaving task aversiveness and mood unchanged, a selective pattern that a general wish to please the experimenter would not readily explain. Nevertheless, self-report cannot confirm that participants engaged exactly as they described, and a supervised or behaviorally instrumented replication would provide direct behavioral confirmation of the pattern observed across the self-report measures.

A third limitation concerns the timing of the mediation analyses. Effort valuation was intended to capture an experience present during the work period, but it was measured after the period ended so that probing it mid-task would not alter how participants appraised their effort in the moment. Willingness to continue was measured earlier, at the mid-task checkpoint. Because the mediator was measured after the outcome, these models cannot establish that effort valuation temporally preceded willingness to continue, and a retrospective rating may partly reflect the full work period rather than the mid-task state alone. We therefore interpret the indirect effects as statistical associations consistent with the proposed mechanism rather than as evidence of temporal mediation. Future designs could capture effort valuation before the persistence outcome without disrupting the task, or measure willingness to continue again after the work period.

Fourth, the single-session design limits conclusions about enduring change. Although reinforcement increased the perceived rewardingness of effort and motivation to persist, the study cannot determine whether these effects reflect transient activation or durable learning. Learned industriousness theory emphasizes gradual changes in effort valuation through repeated reinforcement (Eisenberger, 1992). Such processes cannot be fully captured within a brief task. Future work should therefore examine repeated exposure to subtask–reward pairings across multiple sessions or days. Longitudinal designs would allow stronger tests of cumulative change in effort valuation, persistence, and procrastination behavior.

Finally, the reinforcement manipulation was intentionally minimal and consisted of brief, symbolic feedback. That a manipulation this small—a few words of on-screen praise—shifted effort valuation and persistence at all is itself notable. However, the corresponding effects were, as expected, modest on the three-item composite—though clearly significant on the exploratory two-item measure—and a larger sample would estimate them more precisely. Additionally, reincluding the participants who did not meet the ±5 min task duration window did not have an impact on the pattern and significance of the condition effects on engagement and persistence (see S1). While this provided a conservative test of reinforcement effects, it remains unclear how variations in reward type, magnitude, or personalization might influence motivation. More salient or individualized rewards may produce stronger or qualitatively different effects, particularly for individuals with entrenched procrastination habits. Systematic variation in reinforcement properties would help identify the minimal conditions under which effort valuation can be altered without undermining intrinsic motivation.

## 5. Conclusions

Returning to the dreaded email or the postponed report, getting started and keeping going are different hurdles, and this study suggests that each has its own remedy. State-level interventions aimed at reducing procrastination represent a growing and promising area of research. The present study contributes to this emerging literature by underscoring the importance of distinguishing between task engagement and task persistence as separate motivational processes that unfold over time. In doing so, it also brings attention to learned industriousness—an understudied theoretical framework in the procrastination literature—as a potential mechanism through which continued effort may become more rewarding. From an applied standpoint, these findings point to the value of simple, low-burden micro-interventions that pair task structuring with immediate reinforcement. Notably, the reinforcement used here was symbolic and low-cost, yet it was associated with increases in how rewarding effort felt and in motivation for continued engagement. Together, these results suggest that small, strategically timed supports embedded in everyday workflows may meaningfully reduce procrastination in real-world settings.

## Figures and Tables

**Figure 1:**
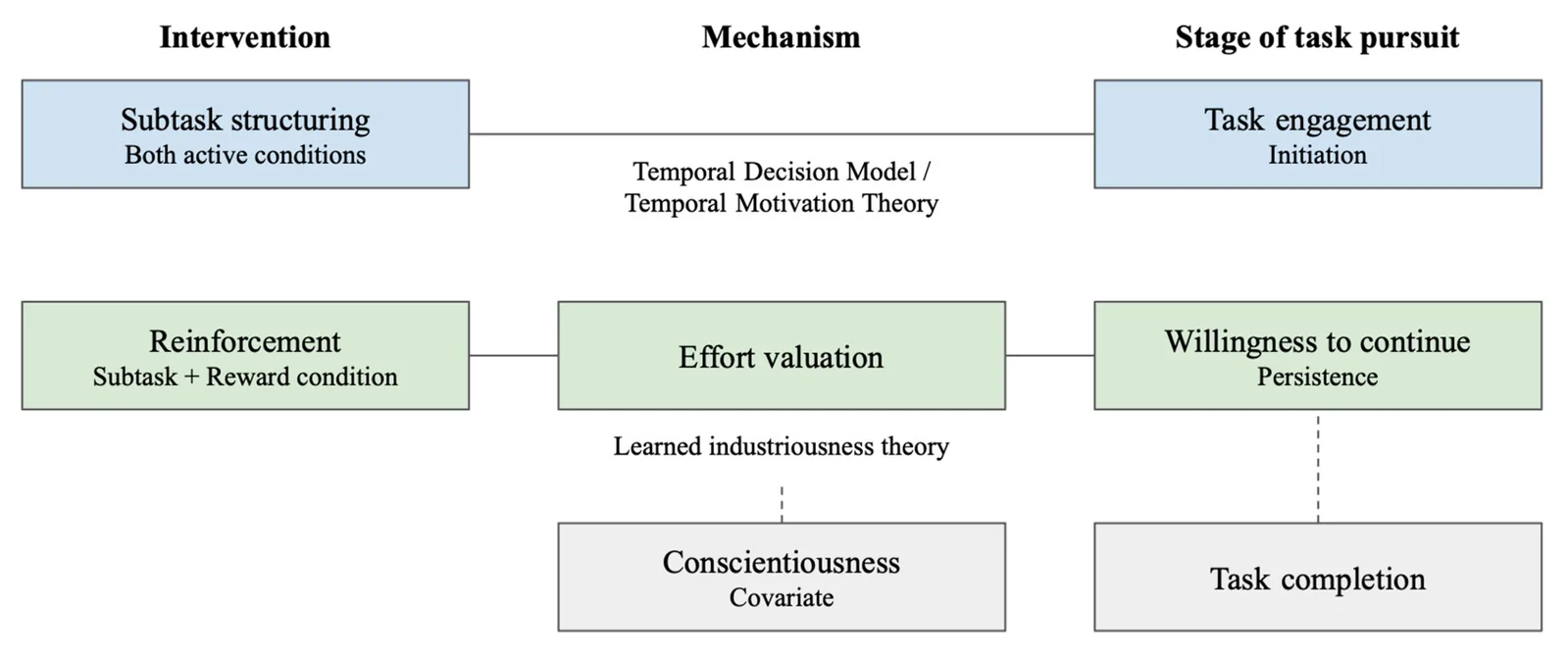
Conceptual model of how subtask structuring and contingent reinforcement influence distinct stages of task pursuit. The model depicts hypothesized relationships tested in the present study. Statistical estimates are reported in Figure 6.

**Figure 2:**
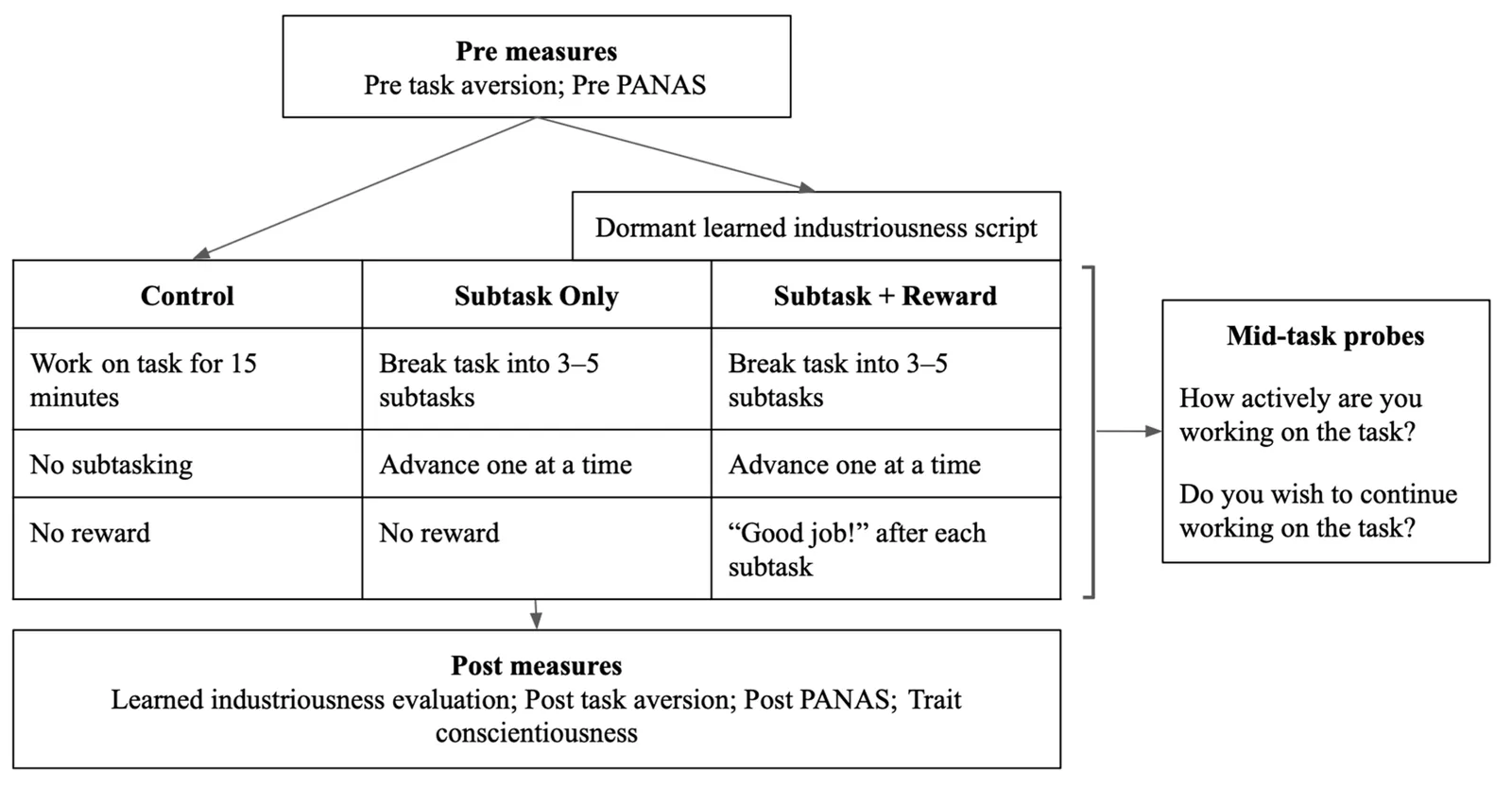
Procedure flowchart.

**Figure 3:**
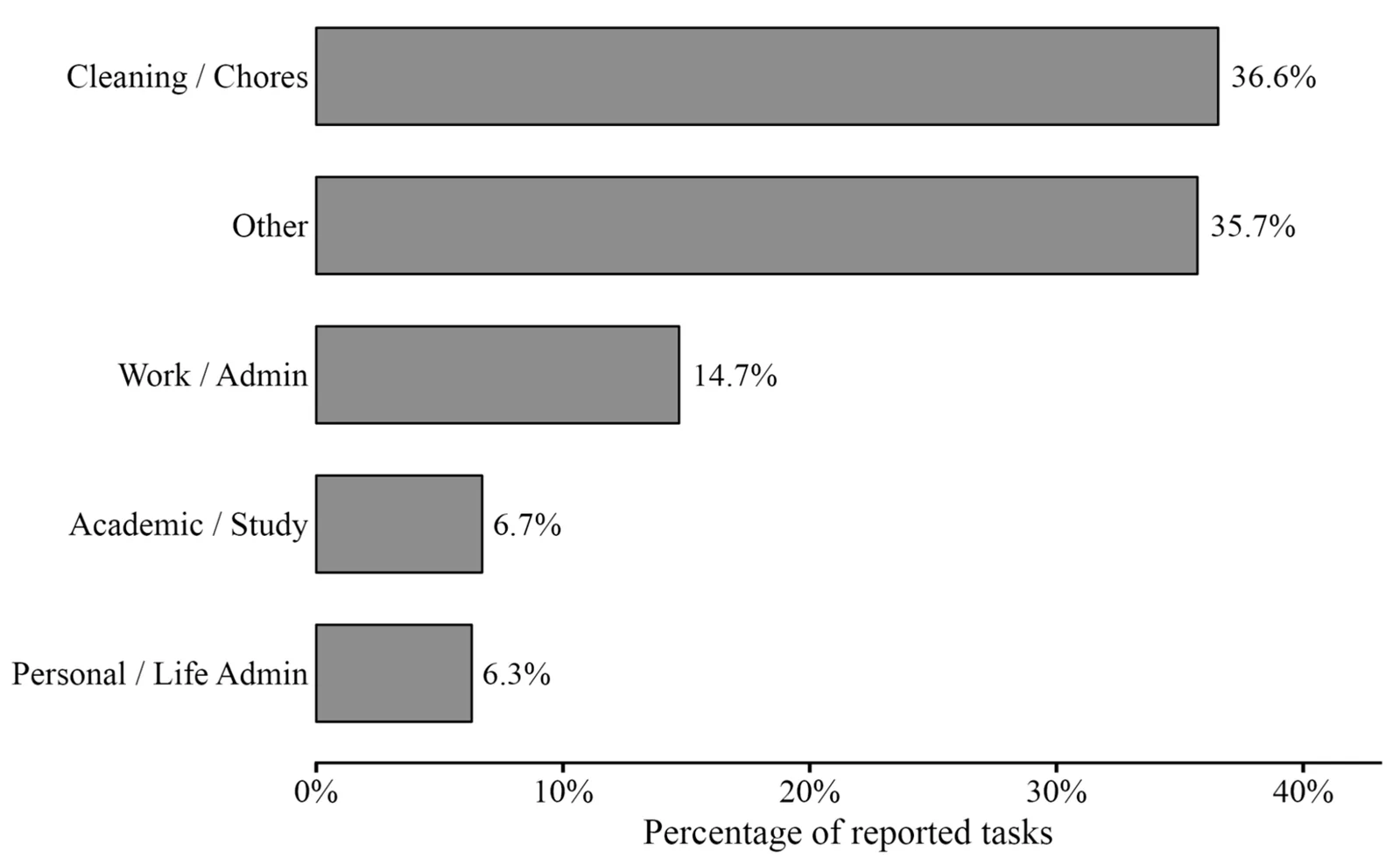
Percentage distribution of self-reported procrastinated tasks across thematic categories. Percentages are calculated as the proportion of total reported tasks within each category.

**Figure 4:**
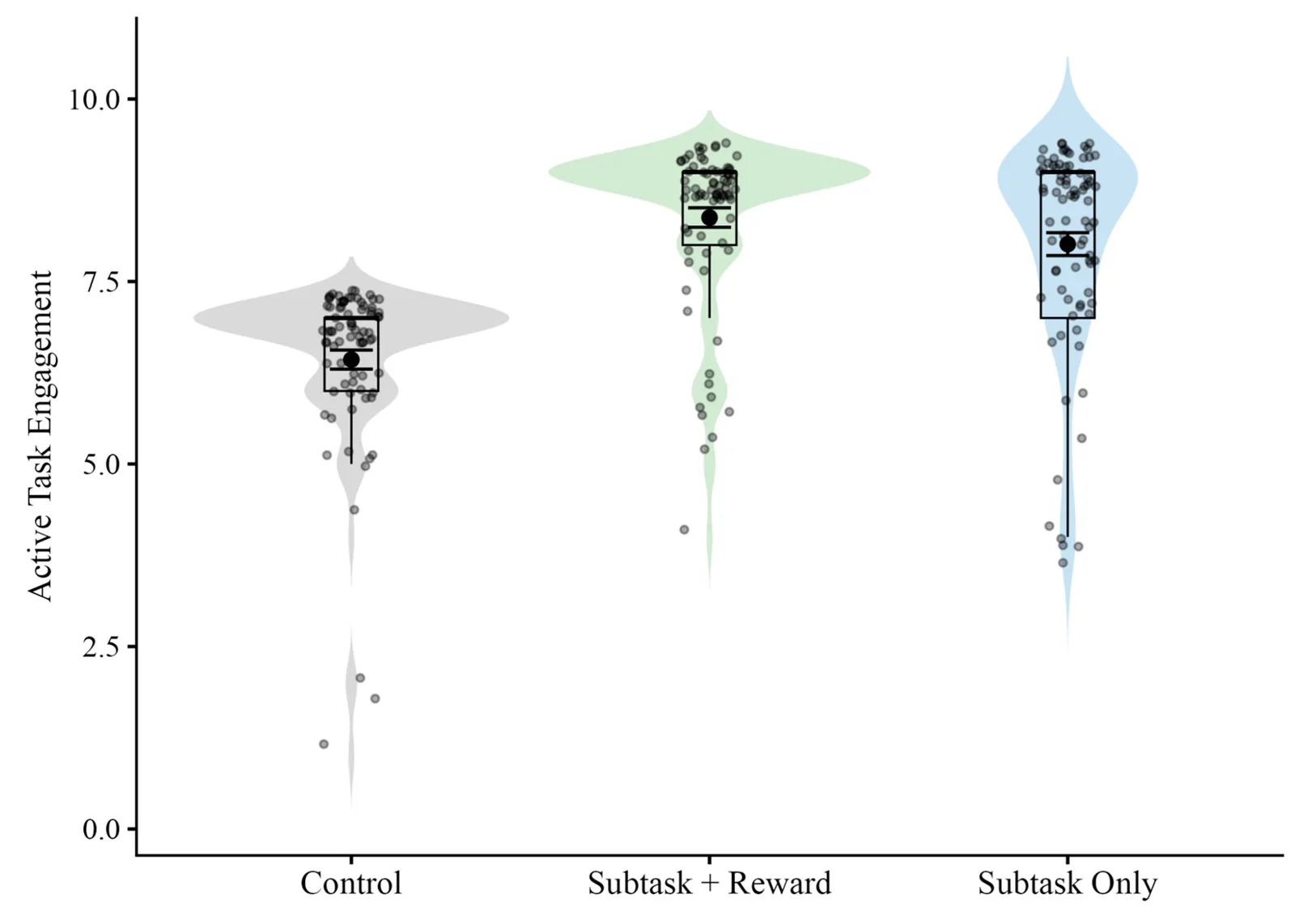
Distribution of self-reported active task engagement at the 8 min interruption point across conditions. Violin plots illustrate the distribution of scores, dots represent individual participants, and overlaid boxplots indicate the median and interquartile range. Error bars reflect ±1 standard error of the mean.

**Figure 5:**
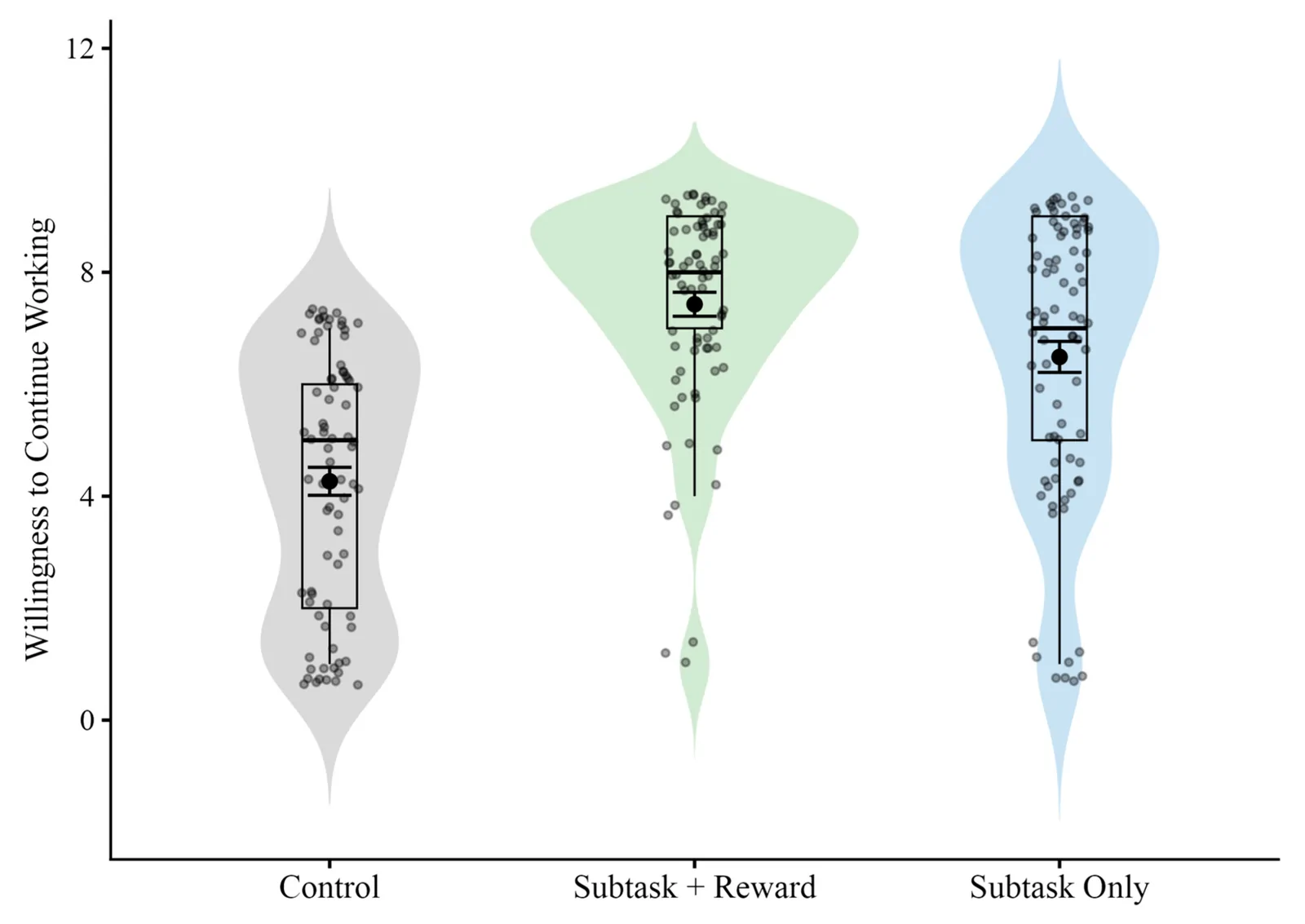
Distribution of self-reported willingness to continue working at the 8 min interruption point across conditions. Violin plots depict the distribution of scores, dots represent individual participants, and overlaid boxplots show the median and interquartile range. Error bars reflect ±1 standard error of the mean.

**Figure 6:**
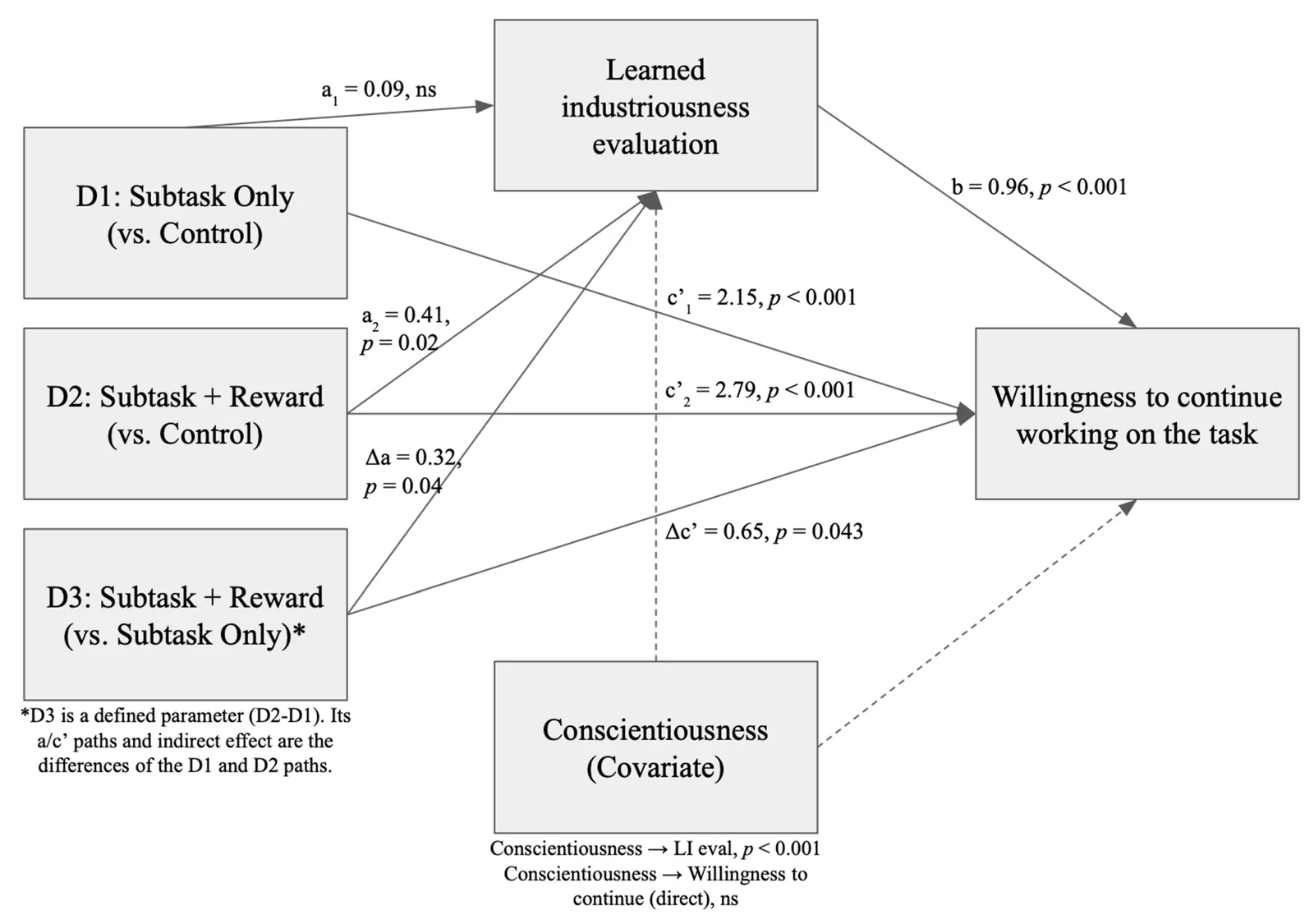
Multicategorical mediation model. D1 and D2 are the two dummy-coded contrasts entered directly into the model; D3 is a defined parameter obtained algebraically as the difference between the D2 and D1 paths. Subtask + Reward significantly predicted learned industriousness evaluation relative to Control; Subtask Only did not. Learned industriousness evaluation significantly predicted willingness to continue across the full sample. The indirect effect was significant for both Subtask + Reward vs. Control, at the significant boundary for Subtask + Reward vs. Subtask Only, and not significant for Subtask Only vs. Control. Direct effects of condition remained significant for all three contrasts. Unstandardized coefficients are displayed. Bootstrap standard errors (5000 resamples, fixed seed = 20260815) were used to estimate indirect effects.

**Table 1:** Correlations among continuous variables.

	1	2	3	4	5	6	7	8	9	10	11	12	13
1. Time estimate	—												
2. Aversion (pre)	0.06	—											
3. Active engagement	0.10	−0.07	—										
4. Willingness to continue	0.08	−0.14 *	0.46 ***	—									
5. Aversion (post)	0.01	0.48 ***	−0.14 *	−0.35 ***	—								
6. PANAS+ (pre)	0.16 *	−0.26 ***	0.17 *	0.28 ***	−0.19 **	—							
7. PANAS- (pre)	0.00	0.27 ***	−0.03	−0.01	0.21 ***	−0.15 *	—						
8. PANAS+ (post)	0.17 **	−0.17 **	0.25 ***	0.42 ***	−0.27 ***	0.81 ***	−0.15 *	—					
9. PANAS- (post)	0.04	0.20 **	−0.04	−0.12	0.36 ***	−0.10	0.77 ***	−0.25 ***	—				
10. Conscientiousness	0.00	−0.14 *	0.13 *	0.12	−0.15 *	0.39 ***	−0.34 ***	0.37 ***	−0.21 ***	—			
11. LI evaluation	0.10	−0.18 **	0.28 ***	0.49 ***	−0.42 ***	0.49 ***	−0.12	0.67 ***	−0.28 ***	0.30 ***	—		
12. PANAS+ change	0.01	0.16 *	0.13 *	0.21 ***	−0.12	−0.36 ***	0.01	0.25 ***	−0.23 ***	−0.05	0.26 ***	—	
13. PANAS- change	0.06	−0.13 *	−0.01	−0.15 *	0.17 **	0.10	−0.49 ***	−0.11	0.18 **	0.24 ***	−0.20 **	−0.34 ***	—
14. Aversion change	−0.05	−0.53 ***	−0.06	−0.19 **	0.49 ***	0.07	−0.06	−0.10	0.15 *	−0.00	−0.23 ***	−0.28 ***	0.30 ***

Note. Pearson correlations; * *p* < 0.05, ** *p* < 0.01, *** *p* < 0.001. Column/row numbers correspond to the numbered variable list above.

## Data Availability

Data is available upon request.

## Supplementary Materials

The following supporting information can be downloaded at https://www.mdpi.com/article/10.3390/bs16101832/s1, Section S1: Sensitivity Analysis Including Time-Window-Excluded Participants; Section S2: Full Mediation Results for the Three-Item and Two-Item Composites; Table S1a: Multicategorical mediation estimates using the three-item learned-industriousness composite. Outcome: willingness to continue working; Table S1b: Multicategorical mediation estimates using the two-item effort-valuation composite. Outcome: willingness to continue working.
