# Supplementary material for: Breaking Procrastination Down: Differential Effects of Subtasking and Reinforcement on Engagement and Persistence
Source: Behav Sci (Basel). 2026 Oct 7;16(10):1832. doi: 10.3390/bs16101832 (PMC13701861; doi:10.3390/bs16101832)
Supplement: Supplementary file 1 [file behavsci-16-01832-s001.zip › behavsci-4488611-supplementary.pdf]

Supplementary Materials

S1. Sensitivity Analysis Including Time-Window-Excluded Participants

The primary analyses excluded 67 participants for reasons related to task duration: participants who reported a task duration outside the intended ±5-minute window around the 15-minute session (i.e., less than 10 minutes or more than 20 minutes), as well as participants who did not report a duration at all. To evaluate whether the ±5-minute window specifically affected the reported results, we reran the primary condition analyses on active task engagement and willingness to continue working after reincluding participants who had reported a valid duration outside that window; participants with no reported duration remained excluded, since there was no value against which to evaluate this criterion. This yielded a relaxed sample of N = 298 (100 Control, 99 Subtask Only, 99 Subtask + Reward) – an increase of 60 participants over the primary sample (N = 238). The remaining 7 of the 67 originally excluded participants had a missing rather than an out-of-window duration value and were therefore not eligible for reinclusion under this relaxed criterion.

Results were consistent with those from the primary analytic sample. Active task engagement again differed significantly by condition, $F(2, 295) = 59.69$, $p < .001$, $\eta^2 = .29$, and willingness to continue working also differed significantly by condition, $F(2, 295) = 55.57$, $p < .001$. The overall pattern and statistical significance of both effects were unchanged relative to the primary analytic sample ($N = 238$), indicating that the ±5-minute time-window exclusion criterion did not drive the reported findings.

S2. Full Mediation Results for the Three-Item and Two-Item Composites

The primary mediation analysis reported in the main text (Section 3.7) used a three-item learned-industriousness evaluation composite. Because one of these items ("I put a lot of effort into this task") indexes effort expenditure rather than the reward value of effort, we additionally examined a two-item effort-valuation composite ("I enjoyed putting effort into this task"; "Being productive during this task felt rewarding") as a sensitivity analysis. This section reports the full parameter estimates for both composites in parallel.

Both models were single multicategorical mediation models estimated in *lavaan*, with condition dummy-coded relative to Control and the Subtask + Reward versus Subtask Only contrast obtained as a defined parameter within the same model. Conscientiousness was included as a covariate on both the mediator and the outcome. Indirect effects were estimated with nonparametric bootstrap standard errors (5,000 resamples; percentile 95% confidence intervals; fixed seed = 20260815). Unstandardized coefficients are reported, and all models were estimated on the full analytic sample (N = 238). For each contrast, a is the condition-to-mediator path, b is the mediator-to-outcome path, ab is the indirect effect, c' is the direct effect, and c is the total effect ($c = c' + ab$).

Table S1a. Multicategorical mediation estimates using the three-item learned-industriousness composite. Outcome: willingness to continue working.

**Contrast**	***a (SE, p)***	***b (SE, p)***	***ab (SE, p)* [95% CI]**	***c' (SE, p)* [95% CI]**	***c (SE, p)***
Subtask Only vs. Control	0.09 (0.19, .640)	0.96 (0.11, < .001)	0.09 (0.19, .645) [−0.26, 0.46]	2.15 (0.30, < .001) [1.54, 2.73]	2.23 (0.37, < .001)
Subtask + Reward vs. Control	0.41 (0.17, .016)	0.96 (0.11, < .001)	0.40 (0.18, .024) [0.08, 0.78]	2.79 (0.29, < .001) [2.18, 3.36]	3.19 (0.33, < .001)
Subtask + Reward vs. Subtask Only	0.32 (0.16, .042)	0.96 (0.11, < .001)	0.31 (0.16, .053) [0.01, 0.65]	0.65 (0.32, .043) [0.02, 1.28]	0.96 (0.35, .006)

Note. Conscientiousness (covariate) → mediator: b = 0.04 (SE = 0.01, p < .001); conscientiousness → willingness to continue (direct): b = 0.00 (SE = 0.01, p = .945).

Table S1b. Multicategorical mediation estimates using the two-item effort-valuation composite. Outcome: willingness to continue working.

**Contrast**	***a (SE, p)***	***b (SE, p)***	***ab (SE, p)* [95% CI]**	***c' (SE, p)* [95% CI]**	***c (SE, p)***
Subtask Only vs. Control	0.16 (0.23, .501)	0.85 (0.09, < .001)	0.13 (0.20, .507) [−0.24, 0.53]	2.10 (0.30, < .001) [1.50, 2.68]	2.23 (0.37, < .001)
Subtask + Reward vs. Control	0.64 (0.19, .001)	0.85 (0.09, < .001)	0.55 (0.18, .002) [0.22, 0.92]	2.64 (0.29, < .001) [2.05, 3.22]	3.19 (0.33, < .001)
Subtask + Reward vs. Subtask Only	0.49 (0.19, .010)	0.85 (0.09, < .001)	0.41 (0.17, .015) [0.10, 0.77]	0.54 (0.32, .086) [−0.08, 1.17]	0.96 (0.35, .006)

Note. Conscientiousness (covariate) → mediator: $b = 0.04$ ($SE = 0.01$, $p < .001$); conscientiousness → willingness to continue (direct): $b = 0.01$ ($SE = 0.01$, $p = .683$).

The two composites yielded the same qualitative pattern. The indirect effect was nonsignificant for the Subtask Only versus Control contrast under both composites and significant for the Subtask + Reward versus Control contrast under both, with a larger indirect effect on the two-item composite ($ab = 0.55$ versus 0.40). For the focal Subtask + Reward versus Subtask Only contrast, the indirect effect moved from the boundary of significance on the three-item composite ($ab = 0.31$, $p = .053$) to clearly significant on the two-item composite ($ab = 0.41$, $p = .015$). Correspondingly, the residual direct effect for this contrast declined from significant ($c' = 0.65$, $p = .043$) to nonsignificant ($c' = 0.54$, $p = .086$), while the total effect was unchanged ($c = 0.96$, $p = .006$). The total effect was identical across composites for every contrast, as expected, because the total effect does not depend on which mediator decomposes it.
